# Effects of 4 weeks of whole-body vibration training on energy expenditure during deep squats of male well-trained students

**DOI:** 10.3389/fphys.2023.1232045

**Published:** 2023-10-11

**Authors:** Xiaohan Huang, Zhengyang Ye, Xuelin Qin

**Affiliations:** ^1^ Education and Training Integration Center, Nanjing Sport Institute, Nanjing, China; ^2^ School of Sports Medicine and Health, Chengdu Sport University, Chengdu, China; ^3^ Department of Physical Education, Gdańsk University of Physical Education and Sport, Gdańsk, Poland

**Keywords:** resistance exercise vibration, resistance exercise, oxygen uptake, energy metabolism, weight-bearing squats

## Abstract

From the perspective of energy expenditure, this study investigated the effects of whole-body vibration training on the energy metabolism of deep squats with different weights. Twenty-two healthy male college students with sports experiences were selected and randomly assigned to perform resistance exercise vibration (REV) or resistance exercise (RE) with varying loads two times per week for 4 weeks. Oxygen uptake and heart rate were measured before, during, and after exercises using a gas analyzer, and energy expenditure was calculated. The results showed the following: 1) the oxygen uptake and energy expenditure of the REV group were significantly higher than those of the RE group during and 30 min after exercise (*p* < 0.01), respectively, and the excess post-exercise oxygen consumption (EPOC) was also significantly higher than that of the RE group (*p* < 0.01). 2) Changes in the oxygen uptake and energy expenditure were stable with increasing exercise in both vibration and non-vibration conditions. There was no difference in energy expenditure per unit of body mass *versus* additional energy per kilogram of body weight (*p* > 0.05). 3) No significant differences in changing exercise intensity were observed in the REV group compared to those in the RE group during the adjacent incremental load phases of △ (40%–0%) and △ (80%–40%) of load during and 30 min after exercise (*p* > 0.05). Our results suggest that 1) vibration training can increase energy expenditure during low-intensity training and excess post-exercise oxygen consumption, and improve the exercise intensity. 2) The effects on energy expenditure were the same for both weight-bearing and non-weight-bearing deep squats, up to 40% of body mass.

## 1 Introduction

At the present stage, whole-body vibration training (Van Heuvelen et al., 2021) has been widely used in the field of sports training and rehabilitation, which is an efficient, fast, non-invasive, and easy-to-use training tool that not only effectively increases muscle strength (Cormie et al., 2006; Bogaerts et al., 2007; Minhaj et al., 2022) and power (Pamukoff et al., 2016) and improves body composition (da Cunha de Sá-Caputo et al., 2021; Rubio-Arias et al., 2021; Reis-Silva et al., 2022) but also rapidly increases energy expenditure (Alizadeh et al., 2020; Jiménez-Pérez et al., 2020). Da et al. (2007) showed that vibration training increased oxygen uptake by 17% compared to non-vibration training. The squat, as an essential compound exercise in strength training, is crucial to the physiological function and athletic performance of athletes (Salmanpour, 2022), which can increase muscle strength (Taheri, 2018), endurance, and explosive power, and also effectively improve the body’s reaction time (Taheri, 2017) and adaptive ability and prevent sports injuries (Al-Azzawi et al., 2023). Therefore, by understanding the effects of vibration training on energy metabolism in squatting (Hibino et al., 2023), we can integrate vibration training into athletes’ strength training programs and the general public’s sports and fitness (Dos Santos et al., 2023), and maximize the optimization of athletes’ training effects and the development of more personalized training programs by enhancing energy expenditure. Meanwhile, Serravite et al. also found, in their study, that oxygen uptake (VO_2_) consumption was significantly greater during high-frequency 20% vibration training than during non-vibration training (Serravite et al., 2008).

The metabolic rate of the body can be increased by vibration training (Cristi-Montero et al., 2013). The study by Dionisio VC and others found that significant changes in body composition occurred with relatively low-intensity vibration training three times a week (Dionisio et al., 2021). It accelerates the effect of fat loss. It also provides a new avenue for sports enthusiasts with fat loss needs. Fat reduction in public sports mainly relies on energy consumption during exercise. The more activity and the longer the exercise time, the more energy the body consumes (Jiménez-Pérez et al., 2020). In a study by DaSilva et al., it was concluded that the vibration group had a higher energy expenditure and RPE than the non-vibration group during the exercise and recovery periods (Da et al., 2007). When the intensity of exercise is higher, the body consumes more and needs more time to recover, and the duration of excess post-exercise oxygen consumption (EPOC) is also longer, indicating that during the recovery process, the body needs to consume more oxygen to return to the resting state (Tsolakis et al., 2020). This is one of the main reasons that EPOC is significantly higher after high-intensity resistive training than lower-intensity resistive training.

Studies suggest that the effect of exercise intensity on EPOC is more important than the time spent exercising (Dionisio et al., 2021). It has been found that exercise intensity can affect the amount and duration of EPOC. However, exercise duration can only affect the duration of EPOC. In addition to exercise intensity and exercise duration, EPOC is also affected by some physiological factors (Cochrane et al., 2008). It has also been shown that there is a linear relationship between oxygen uptake and amplitude, which makes it clear that regardless of population, type of exercise, or whether it is weight-bearing or not, as long as the amplitude changes, oxygen uptake will change and metabolic capacity may not increase, but greater energy expenditure can be realized (Rittweger et al., 2001). There is also the removal of lactic acid from muscle tissue during exercise, the hemoglobin load in blood and the myoglobin load in muscle tissue, oxygen load, oxygen load of myoglobin in muscle tissue, gas exchange, body temperature, heart rate, and hormone maintenance at high levels (Gomes-Neto et al., 2021).

The purpose of this experimental study was to investigate the difference in energy expenditure between the resistance exercise vibration (REV) and resistance exercise (RE) groups under different weighted squatting conditions (Dionisio et al., 2021). Does vibration training alter energy expenditure during deep squats? Does vibration training have different effects on energy metabolism under different weight-bearing conditions? Experiments by Bertucci W M and others demonstrated that in terms of energy expenditure and metabolic equivalent values, energy expenditure was significantly greater with REV than with RE, but energy expenditure was greater for low amplitude than for high amplitude and no vibration (Bertucci et al., 2015).

There are two main intervention factors in this experiment: one is the mechanical factor. According to Rittweger J et al., 30 Hz is a relatively suitable frequency stimulus for the human body during vibration training (Rittweger et al., 2001). Bertucci believes that the experimental results in the case of frequency 30 Hz are average and that 50 Hz should be used for best results (Bertucci et al., 2015). The conventional parameter selection is about 26–45 Hz and 2∼6 mm; and too low and too high vibration stimulation have some adverse effects on the human body, so our research parameters are constant in the mechanical vibration amplitude of 2 mm and vibration frequency of 30 Hz. The other factor is the external resistance load change factor. Three loads were selected in the study by Serravite et al., which were 0, 20, and 40% of body weight (Serravite et al., 2008); meanwhile, in our study, the loads were finally set at 0, 20, 40, and 80% in order to try to understand more about the effect of loads on energy consumption.

In this study, our results were statistically analyzed to demonstrate that vibration stimulation can increase additional energy consumption and deepen EPOC; with the increase in load, the intensity of energy consumption increased by vibration training is more than that increased by non-vibration training (Avelar et al., 2011). On the other hand, vibration training can increase the energy metabolic rate and ability to oxidize sugar during exercise, thus achieving the effect of accelerating fat loss (Rittweger et al., 2001). Therefore, for the athletic training and mass fitness fat loss crowd, this can also serve as an efficient, more scientific, and effective training guide.

## 2 Materials and methods

### 2.1 Experimental approach to study

According to previous studies (Garatachea et al., 2007), when the frequency is below 20 Hz, the resonance phenomenon with each organ can be easily triggered, which will cause harm to the human body. However, the conventional frequency choice for vibration training is 30 Hz, which proved to be a relatively appropriate body frequency stimulus (Rittweger et al., 2001), and the choice of the 2-mm amplitude exercise condition was a more appropriate intervention stimulus, with the lower amplitude producing more EE (energy expenditure) and RPE (respiratory exchange rate) compared to the higher amplitude of 4 mm (Serravite et al., 2008). So, in our experiment, the vibration frequency is set at 30 Hz, the amplitude is set at 2 mm, and the frequency of each vibration training is about 3 days (Rehn et al., 2007). We randomly sorted each person before the experiment into two groups, vibration and no vibration, respectively, according to body mass 0, 20, 40, and 80% of the load. A total of eight experiments were conducted, comprising a total experimental period of about 4 weeks, while the data before and after the experiment were used to compare and analyze.

### 2.2 Subjects

To calculate the sample size for the experiment, we used G*Power software. We set one tail, effect size to 0.5, *α* to 0.05, and power to 0.8. We calculated that we needed to recruit at least 21 subjects. Therefore, we selected 22 healthy male college students as subjects (Table 1), and the Research Ethics Committee of Nanjing Sport Institute approved this study protocol (registration number RT-2023-08). Participants in this study must meet the following criteria: 1) have at least 3 years of exercise experience; 2) have no history of cardiovascular disease, injury, or discomfort during vibration stimulation training; and 3) be able to perform at least 10 repetitions of squats at body weight. We asked them to refrain from other strenuous exercises for 2 days before each experiment to avoid interference with the results of the experiment, and they had to sign a consent form.

**TABLE 1:** Basic information of subjects (data indicated by mean ± SD).

Number of people (persons)	Age (year)	Height (cm)	Body mass (kg)	BMI (kg/m^2^)
22	23.8 ± 1.6	177.3 ± 4.31	73.3 ± 3.74	21.86 ± 1.60

### 2.3 Training program and prescription

In order to prevent stress reactions during the formal experiment, 1 week before the start of the experiment, each subject was subjected to shaking table and oxygen mask use, and pre-experimental simulations were conducted to further familiarize them with the whole process of the experiment. To reduce the effect of abnormal food thermogenesis and resting metabolism on the experimental results, 6–8 h of adequate sleep was ensured, eating should be completed 2 h before the start of the experiment, and the time of each experiment was consistent with the pre-experimental diet.

**FIGURE 1:**
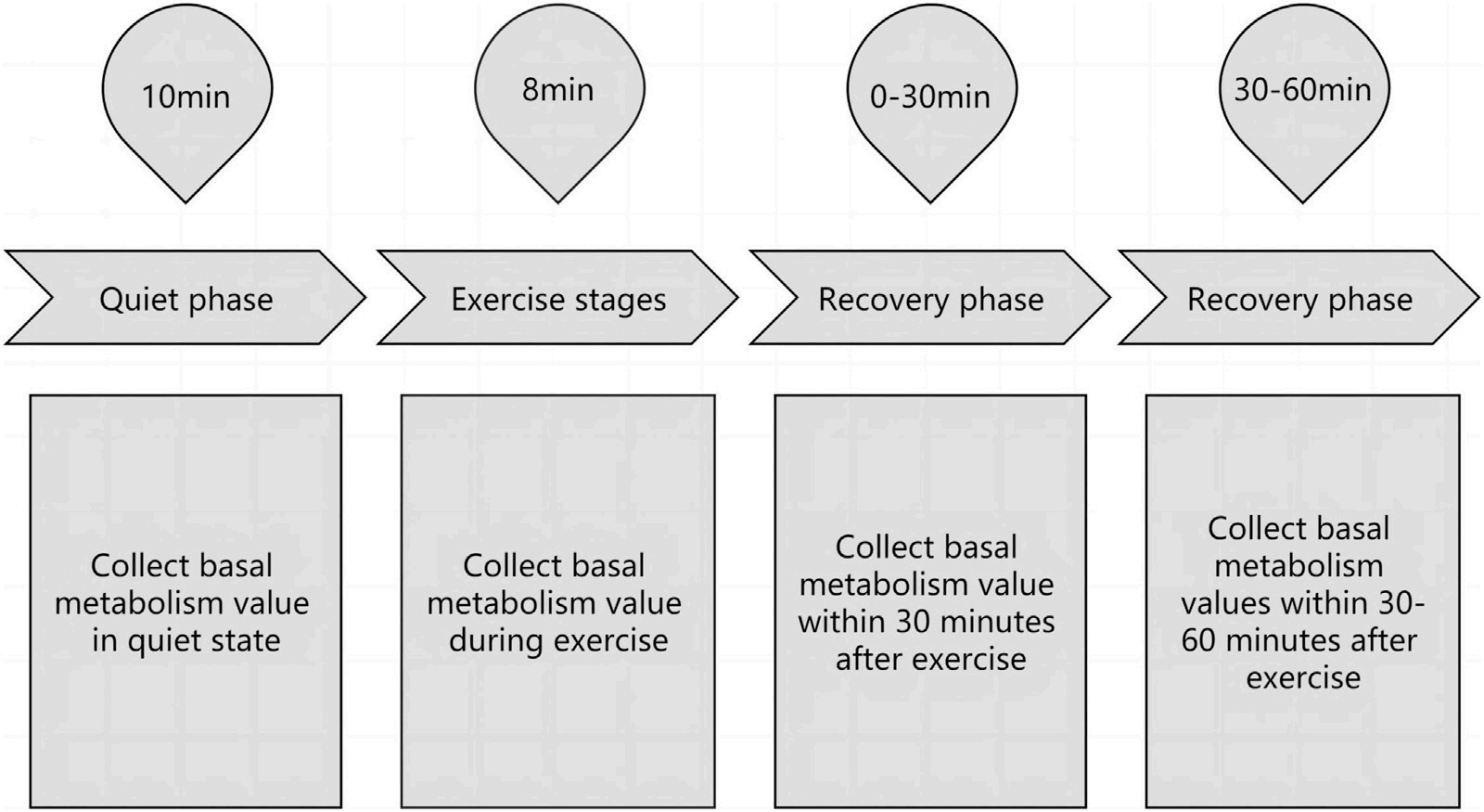
Schematic diagram of an experimental process.

We used the Power Plate vibration trainer. Before each experiment, the participant entered the laboratory, sat and rested, wore an oxygen mask, and was monitored using a cortex gas metabolism analyzer. When stabilized to basal metabolism, it was recorded in the resting condition 10 min before exercise and in exercise conditions. Six groups of deep squats were performed, 10 per group, according to the rhythm of the metronome, 3 s every time, with each group interval of 60 s (Van Heuvelen et al., 2021). Immediately after exercise, the basal metabolic values were collected during the complete return to the resting condition as a complete experimental procedure (Figure 1).

The formula (Goudarzian et al., 2021) is then used to calculate the total energy expenditure during and after the exercise.

CHO = (4.585*CO_2_) − (3.2255*CO_2_)2
FAT = (1.6946*CO_2_) − (1.7012*CO_2_)2
EE = (CHO*4) + (FAT*9)

To obtain the actual energy expended during exercise, the energy produced by resting metabolism during exercise (EE-REE) was subtracted from the total energy expenditure in each phase (Alizadeh et al., 2020).

REE = 88.362+(13.397*body weight)+(4.799*height)−(5.677*age)

### 2.4 Statistical analysis

SPSS 18.0 was used for statistical analysis, and results were expressed as mean ± standard deviation (X ± SD). To test whether energy expenditure, oxygen uptake, and other indices were statistically different under different loads in the vibration and non-vibration conditions, two-factor repeated measures ANOVA was used. Whenever two factors interacted, separate effects were identified, and when they did not, the main effects were identified and effect sizes were reported to highlight the separate effects. Each group’s data were tested by Shapiro–Wilk’s method and the *p*-value was greater than 0.05, indicating that they followed a normal distribution. The *p*-value of Levene’s chi-square test was greater than 0.05, and the data were consistent with equal variance. Then, according to the sphericity test, if the *p*-value is greater than 0.05, it means that there is no correlation between the data on repeated measurements. If the *p*-value was less than 0.05, the correction was performed with Greenhouse–Geisser correction. The statistically significant level was *p* < 0.05. The highly significant level was *p* < 0.01. Then, we defined the “∆” notation to indicate the change in relative oxygen uptake between two different loading levels. For example, ∆ (40%–0%) denotes the change in relative oxygen uptake between 0% and 40% loads, while ∆ (80%–40%) denotes the change in relative oxygen uptake between 40% and 80% loads.

## 3 Research results

### 3.1 Changes in oxygen uptake during different phases of vibration training

**FIGURE 2:**
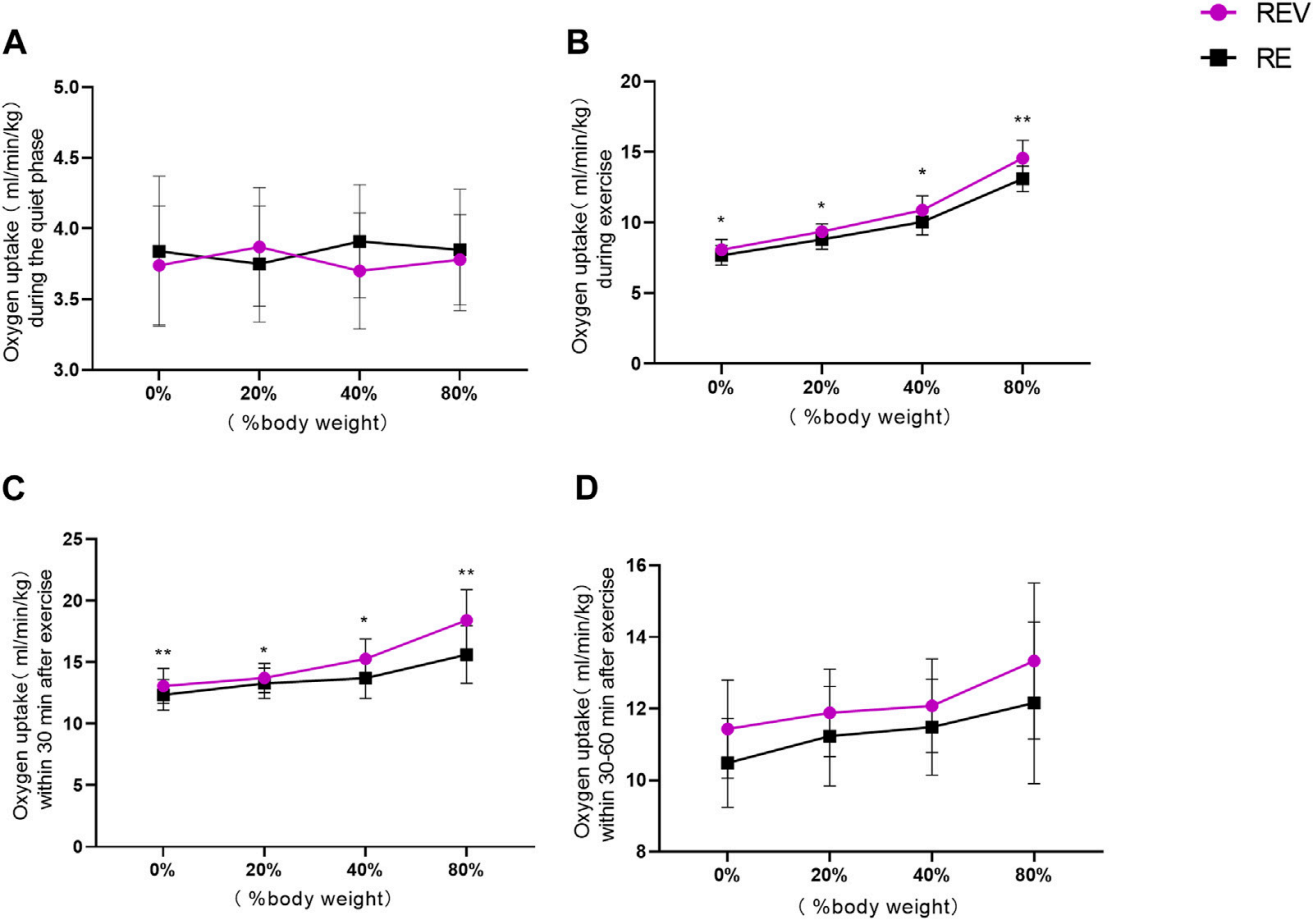
Changes in oxygen uptake during various stages of exercise (*p < 0.05; **p < 0.01).

Statistical analysis of the changes in oxygen uptake in different phases of the exercise process and the reliability of its data was conducted, according to confidence intervals (Roelants et al., 2004). Total oxygen uptake in 10 min during the rest phase before exercise, “vibration stimulation” (F = 2.496, *p* = 0.149), “load” (F = 0.018, *p* = 0.997), and no difference obtained from a statistical test due to “vibration stimulation” * “load” *p* = 0.143 is shown in Figure 2.

Total oxygen uptake during exercise in 8 min, “vibration stimulation” (F = 31.972, *p* = 0.000), “load” (F = 170.784, *p* = 0.000), and by “vibration stimulation"*"load” (F = 3.896, *p* = 0.020) had an interaction effect according to a statistical test (*p* < 0. 05). The oxygen uptake of the body gradually increased with the increase in load in both REV and RE groups, respectively, and there was a significant difference (*p* < 0.01) between the groups (Table 2).

**TABLE 2:** Analysis of the difference in oxygen uptake between the two phases with significant differences.

Weight (%)	Campaign phase	Difference in oxygen uptake (L)	*p*-value	95% confidence interval
0	8 min in motion	2.314	0.023	0.404–4.223
	30 min after exercise	4.325	0.000	2.651–5.999
20	8 min in motion	3.344	0.031	0.387–6.301
	30 min after exercise	8.344	0.013	0.387–6.301
40	8 min in motion	4.990	0.019	1.047–8.934
	30 min after exercise	9.454	0.011	2.785–16.123
80	8 min in motion	8.833	0.001	4.413–13.252
	30 min after exercise	13.714	0.000	8.304–19.123

Total oxygen uptake within 30 min after exercise, “vibration stimulation” (F = 49.503, *p* = 0.000), “load” (F = 25.805, *p* = 0.000), and due to “vibration stimulation“*”load” (F = 6.203, *p* = 0.002) had an interaction effect, according to a statistical test (*p* < 0.05). Under the premise of controlling vibration stimulation, the oxygen uptake of the organism gradually increased with increasing load in both REV and RE groups, and there was a significant difference between them (*p* < 0.01). This indicates that vibration stimulation has an effect on oxygen uptake, and the oxygen uptake at 80% load was significantly greater than that at 40% and 20%.

Total oxygen uptake 30–60 min after exercise, ‘vibration stimulation’ (F = 15.810, *p* = 0.003), ‘load’ (F = 8.284, *p* = 0.000), and by ‘vibration stimulation‘*’load’ (F = 0.390, *p* = 0.761) had no interaction effect, according to a statistical test (*p* > 0.05). After 30 min since the end of the exercise, there was no significant difference in the oxygen uptake at 0%, 20%, 40%, and 80%, indicating a basic return to a resting state and the end of EPOC.

### 3.2 Changes in total energy consumption under different phases of vibration training

**FIGURE 3:**
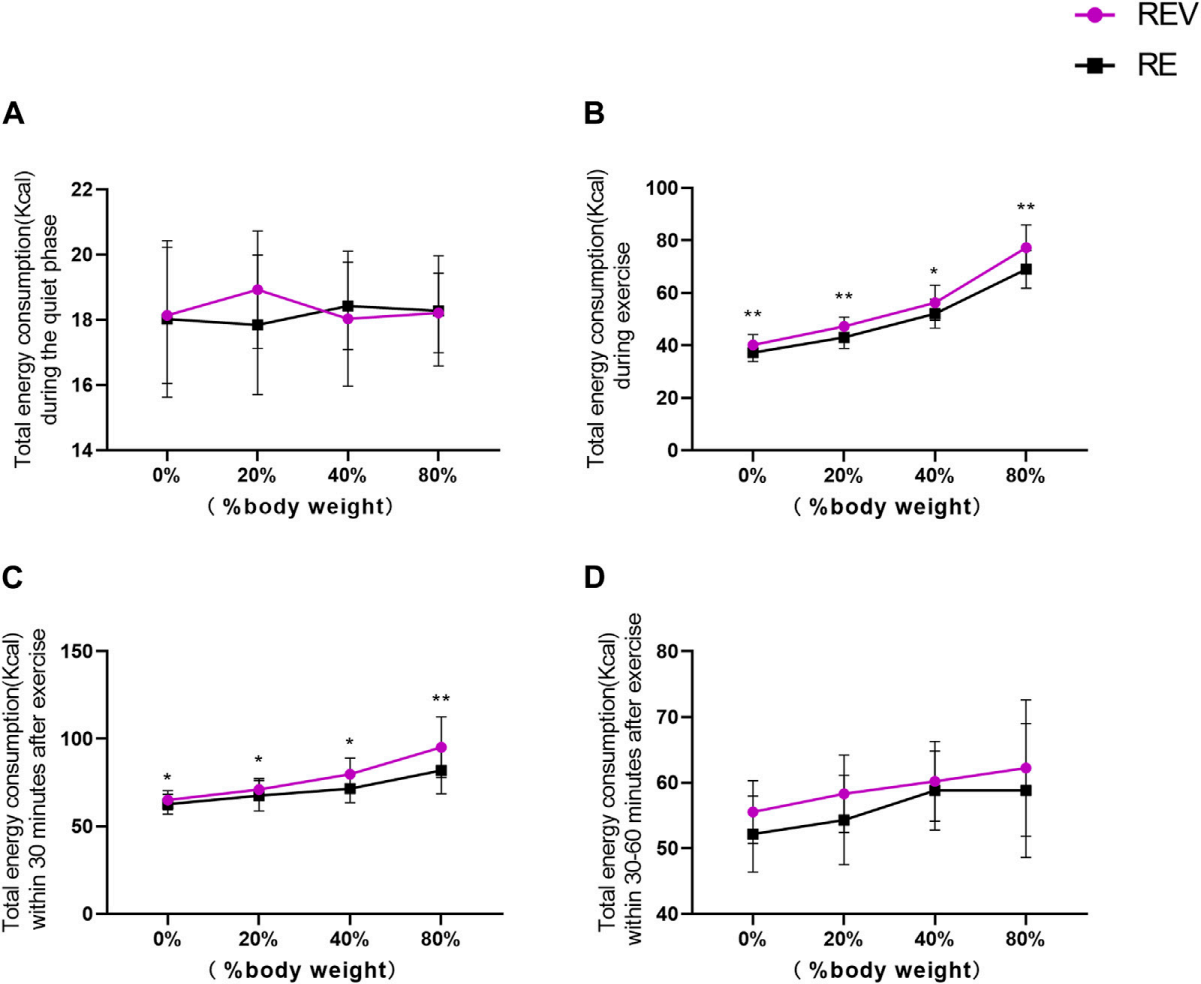
Changes in total energy consumption during different stages of exercise (*p < 0.05; **p < 0.01).

Statistical analysis of total energy expenditure in different phases of the exercise process was performed (shown in Figure 3). The total energy expenditure in the resting phase for 10 min before exercise, “vibration stimulation” (F = 1.126, *p* = 0.316), “load” (F = 0.157, *p* = 0.924), and by "vibration stimulation” * “load” (F = 1.393, *p* = 0.266) was not different according to a statistical test. This indicates that during the resting phase, all groups were at the same basal metabolic level as each other, and therefore, there was no significant difference.

Total energy expenditure during exercise for 8 min, “vibration stimulation” (F = 55.538, *p* = 0.000), “load” (F = 171.713, *p* = 0.000), and due to “vibration stimulation“*”load” (F = 3.012, *p* = 0.047) had an interaction effect according to a statistical test (*p* < 0.05). The energy expenditure of the body gradually increased with the increase in load in both REV and RE groups, respectively, and there was a significant difference between them (*p* < 0.01) (Table 3).

**TABLE 3:** Analysis of the difference in energy consumption between the two phases with significant differences.

Weight (%)	Campaign phase	Energy consumption difference (kcal)	*p*-value	95% confidence interval
0	8 min in motion	17.650	0.009	5.554–29.746
	30 min after exercise	14.071	0.018	3.028–25.114
20	8 min in motion	27.824	0.002	13.488–42.159
	30 min after exercise	21.171	0.044	0.767–41.575
40	8 min in motion	25.010	0.031	2.924–47.096
	30 min after exercise	49.723	0.014	12.805–86.641
80	8 min in motion	49.639	0.001	28.252–71.026
	30 min after exercise	79.545	0.003	36.742–123.348

Total energy expenditure within 30 min after exercise, “vibration stimulation” (F = 31.508, *p* = 0.000), “load” (F = 23.754, *p* = 0.000), and due to “vibration stimulation“*”load” (F = 5.04, *p* = 0.007) had an interaction effect according to a statistical test (*p* < 0.05). The energy expenditure of the body gradually increased with the increase in load in both REV and RE groups, respectively, and there was a significant difference between them (*p* < 0.01). It indicates that the effect of high-intensity vibration exercise is significantly greater than that of low- and medium-intensity exercise.

Total energy expenditure 30–60 min after exercise, “vibration stimulation” (F = 15.837, *p* = 0.003), “load” (F = 5.986, *p* = 0.003), and by “vibration stimulation” * “load” (F = 0.986, *p* = 0.422) had no interaction effect, according to a statistical test (*p* > 0.05). It means that vibration training with different weights after 30 min of exercise did not meet statistical differences in energy expenditure.

### 3.3 Differences in oxygen uptake and energy expenditure between the REV and RE groups during incremental loading

**FIGURE 4:**
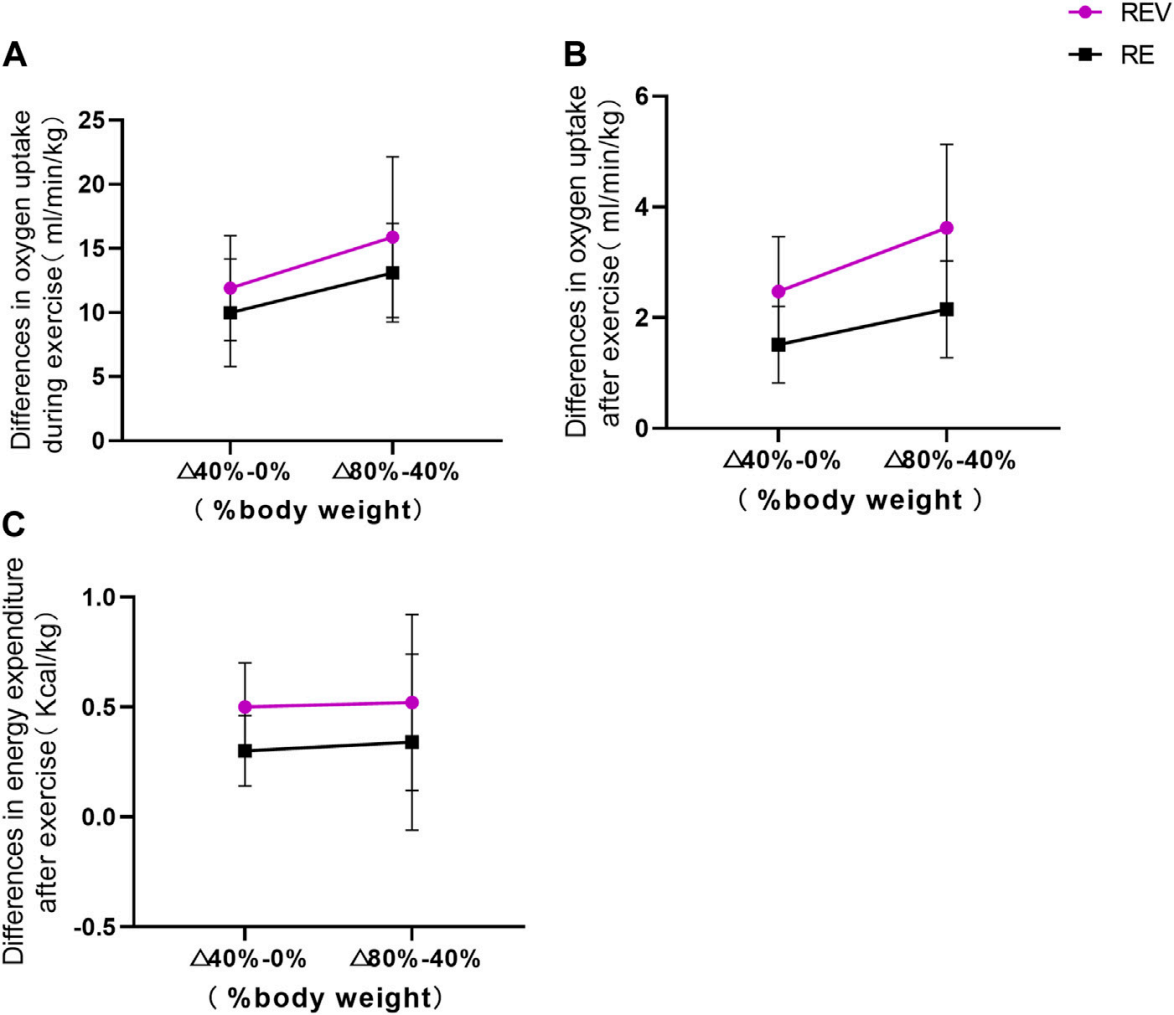
Changes in oxygen uptake and energy consumption during progressive loading (*p < 0.05; **p < 0.01).

To investigate the change in relative oxygen uptake between adjacent incremental loads during exercise (shown in Figure 4), a paired-samples *t*-test was performed in both the △ (40%–0%) and △ (80%–40%) incremental load phases, and the statistical results showed no significant difference between the two groups (*p* > 0.05). When comparing within the groups, the REV (*p* = 0.263) and RE groups (*p* = 0.219) showed no significant effect of the “weight” factor on the relative oxygen uptake (*p* > 0.05). When comparing between the △ (40%–0%) (*p* = 0.135) and △ (80%–40%) groups (*p* = 0.128), there was no significant difference in relative oxygen uptake by “vibration stimulation” (*p* > 0.05). With the increase in load and the application of vibration stimulation, the intensity of the change in oxygen uptake was not significant.

To investigate the change in relative oxygen uptake between adjacent incremental loads after exercise, a paired-samples *t*-test was performed in both the △ (40%–0%) and △ (80%–40%) incremental load phases, and the statistical results showed no significant difference between the two groups (*p* > 0.05). When comparing within the groups, the REV (*p* = 0.303) and RE groups (*p* = 0.559) showed no significant effect of the “weight” factor on the relative oxygen uptake (*p* > 0.05). Comparison between the △(40%–0%) (*p* = 0.134) and △(80%–40%) groups (*p* = 0.157) showed no significant difference between “vibration stimulation” and relative oxygen uptake (*p* > 0.05).

To investigate the change in relative energy expenditure between adjacent incremental loads after exercise, a paired-samples *t*-test was performed in the incremental load △ (40%–0%) and △ (80%–40%) phases, and the statistical results showed that there was no significant difference between the two groups (*p* > 0.05). When comparing within the groups, the REV (*p* = 0.846) and RE groups (*p* = 0.772) showed that the “weight” factor did not have a significant effect on relative energy expenditure (*p* > 0.05). When comparing between the △ (40%–0%) (*p* = 0.055) and △ (80%–40%) groups (*p* = 0.207), it showed that there is no significant difference between “vibration stimulation” and relative energy expenditure (*p* > 0.05).

## 4 Discussion

### 4.1 The effect of weighted vibration training on the body’s oxygen uptake

The purpose of this study was to investigate the effect of whole-body vibration training on energy metabolism during deep squats of different intensities from the perspective of energy expenditure. Our results showed that vibration training can increase energy expenditure and post-exercise excess oxygen consumption during low-intensity training and improve exercise intensity.

Related experimental results show that the average VO of individuals performing resistance half-squat training with vibration stimulation intervention increases by 4.5 mL/kg compared to non-vibration training, which corresponds to 20 Kcal of energy from oxygen consumption and is approximately equal to the exercise intensity at a walking speed of 0.4 m/s, while increasing with vibration frequency, amplitude, and weight (Dallas et al., 2015). In addition, studies have shown that VO_2_ of the REV group was significantly higher than that of the RE group (Serravite et al., 2008), which were 16.6% and 18.9%; it indicates that vibration has an effect on oxygen uptake by stimulation, and the effect of higher-intensity vibration exercise is significantly better than that of low-intensity exercise. When resistance vibration training was performed under different load conditions, VO_2_ of the REV group was also higher than that of the RE group by about 17% (Bertucci et al., 2015). During the phase of weight-bearing exercise, VO_2_ of the REV group during the training period was also significantly higher than that of the RE group because the intensity of the vibration weight-bearing squatting was significantly higher than that of normal squatting; at the same time, the paper corresponds to Serravite’s experiment, on the basis of which the subjects involved were male college students with athletic experience and all of them had proficient experience in squatting practice (Serravite et al., 2008), which could minimize the error of the experiment due to the differences in movements and individual athletic ability issues, thus minimizing the experimental error. At four different loads (0%, 20%, 40%, and 80%), the increase in oxygen uptake in the REV group compared to that in the RE group was 9%, 12%, 8%, and 12%, respectively.

The results of the study by Milanese demonstrated that vibration training may be beneficial in increasing the energy metabolism rate during low weight-bearing deep squats (Milanese et al., 2018). However, the results of this experiment showed that in the △ (40%–0%) and △ (80%–40%) phases, although the mean of the REV group was higher than that of the RE group, there was no difference in oxygen consumption per unit body weight *versus* extra per kilogram at low and medium intensities, resulting in a non-significant difference in the trend of intensity change between REV and RE in incremental loading.

Our study also found that the recovery period becomes longer after a certain intensity of resistance exercise; during the recovery period, high-intensity exercise requires more fat oxidation for energy than low-intensity exercise (Osterberg and Melby, 2000); therefore, oxygen uptake increases. A study also found that vibration training increased the level of lactate in the blood after a deep squat exercise without increasing the exercise load (Amasay et al., 2020; Miokovic et al., 2019). Similarly, studies have also shown that vibration training significantly reduces body fat and insulin levels, which is expected to improve metabolic health, which is very beneficial for the body (Bautmans et al., 2005; Zeigler and Swan, 2016).

The results of this experiment showed that VO_2_ was significantly higher in the REV group than in the RE group in the 0–30 min phase at the end of the post-exercise period, but there was no significant difference in the results in this phase from 30 min to 60 min. The reason for this is that this experiment is different from most experiments because the load selection is lighter and not designed according to 1 RM proportional weight bearing, but selecting small- and medium-intensity loads proportional to body weight for training, coupled with the fact that the subjects are experienced in sports and have a higher recovery ability, so the difference is not significant. There are also differences in gravitational acceleration generated by different shakers during vibration, and the mass of the human body and the external load on the vibration platform are also different. These differences affect the intensity of training, resulting in differences in oxygen uptake and EPOC. Another point is that at the end of vibration training with increasing load, no significant difference was found in the change in oxygen uptake between the REV and RE groups, and the change in exercise intensity tended to be the same after two incremental exercise phases, △ (40%–0%) and △ (80%–40%). This may be because the subjects were a group of people who were experienced in exercise and had a high recovery capacity of the body. Therefore, different individuals have different sensitivities to the load stimulus due to the difference in the strength level, which is also an important factor affecting the experimental results.

### 4.2 The effect of weighted vibration training on the body’s energy expenditure

The main finding of this experiment is that the trend of energy expenditure is stable in each weight-bearing relative energy expenditure and does not cause a difference between the energy consumed per unit of body weight and the additional energy consumed per kilogram. The amount of energy consumed per kilogram per unit of body weight and external load is approximately equal, whether under vibrating or non-vibrating conditions. From this, it can be found that in addition to the energy generated by the active muscle firing contraction during work, there is also a large amount of energy consumption generated by other muscle groups in static contraction to maintain body stability (Hazell and Lemon, 2012). Because the process of standing force involves the muscles of the entire body, indicating that the muscles are continuously contracting, the tibialis anterior, rectus femoris, gluteus maximus, and hamstrings of the extensor group are very active (Rittweger et al., 2000; Patrícia et al., 2021), and the muscles around the calf are mainly contracted statically, as a form of stable support to connect the ground to the body. The energy consumed by the contraction of the stabilizing muscles is not less than that of the active muscles as the plantar flexors, femoral, iliopsoas, and longissimus muscles are highly active during the force process, indicating that these stabilizing muscles also play an important role in postural control (Lima et al., 2020).

The experimental results showed that the energy expenditure of the REV group was significantly higher than that of the RE group. The experiments concluded that the energy expenditure of continuous weighted deep squats with 40 Hz vibration training at 50% of the weighted body weight was greater for 2 mm amplitude than for no amplitude and at 4 mm amplitude (Bertucci et al., 2015). Therefore, it is not necessary to increase the intensity of the vibration to improve energy expenditure during exercise. In other studies, it was also found that the energy expenditure of the vibration group was greater than 15% compared to the non-vibration group during 30 min of continuous dynamic vibration exercise (Vissers et al., 2009). In addition, we found that the energy expenditure of the vibration group was also significantly higher than that of the non-vibration group at 30% of body weight during 4 mm, 30 Hz vibration training (Rittweger et al., 2001).

The results of the present experiment are also in line with the conclusion of the aforementioned authors’ that as the weight becomes heavier, the intensity of change in the energy consumed by the body becomes greater (Dionisio et al., 2021). Even under vibration intervention, it cannot be contrary to common sense. However, the results of the present experiment showed some differences in the loads △ (40%–0%) and △ (80%–40%) REV groups compared to the RE group in the two increasing phases of loads 40%–0% and 80%–40%, but no significant differences were found after statistical tests. So, we inferred that, on one hand, the energy expenditure due to the additional load caused by the vibration stimulation was not as high as expected. On the other hand, individuals with a high level of strength and coordinated movement completion will use less energy compared to others. In particular, at low and medium intensities, the magnitude of the change in energy expenditure with incremental load was also not significant.

### 4.3 Effect of post-exercise vibration on energy expenditure with different weights

Regarding the effect of energy expenditure, researchers found that the REV group had significantly higher energy expenditure than the RE group (Tsolakis et al., 2020). In this study, it was found that the total energy expenditure in the REV group was significantly greater than that in the RE group during and 0–30 min after exercise (Da et al., 2007; Osterberg and Melby, 2000). During phases of the exercise and at the end of the exercise, the energy expenditure of the REV group was significantly higher than that of the RE group. The reason for this is that the intensity of vibration training is greater than that of single resistance training, and it further increases energy expenditure (Alizadeh et al., 2020). Over time, there was no significant difference in energy expenditure in the REV group compared to that in the RE group at the end of the 30–60-min exercise phase (Jiménez-Pérez et al., 2020), suggesting that 1 hour is sufficient to restore the metabolic capacity of the function in low-to-moderate intensity training (Torvinen et al., 2002; Sitjà-Rabert et al., 2012).

From our experimental results, it is found that as the load weight increases, the energy expenditure also increases (Minhaj et al., 2022), but the trend of energy expenditure change intensity does not change much in the REV group compared with that in the RE group as the load increases. The reason for this is probably due to the arrangement of load intensity, where the load is too low, which reduces the intensity of training and is not conducive to deepening the post-exercise excess oxygen consumption, so there is not much difference between the REV and RE groups in terms of the post-exercise change in energy consumption. It shows that during training, a bigger load is not better (Lee and Kim, 2020), and the results are not always accurate when trying to increase the energy expenditure by increasing the load. It should be explored which load interval causes the individual to consume the most energy to improve the training efficiency.

### 4.4 Research limitations

First, the generalizability to different populations and training levels is limited by the relatively small sample size of this study. Further research could be conducted by increasing the sample size and including more subjects from different backgrounds to better understand the effects of whole-body vibration training in different populations. Second, the duration of this study was relatively short. It only covered certain training cycles. Future studies may consider designing interventions of longer duration to understand the ongoing effects of long-term training in order to more fully evaluate the effects of whole-body vibration training. Additionally, measurements of physiological metrics, such as energy expenditure and excess oxygen consumption, were limited in this study. The effects of whole-body vibration training on other physiological measures and athletic performance, such as cardiorespiratory fitness, strength performance, and musculoskeletal adaptations, could be further investigated in future studies. Finally, only one specific vibration parameter and load variation was addressed in this study. Further studies could investigate the effects of different vibration parameters and loading modes on energy metabolism and training effects. This would help optimize whole-body vibration training protocols.

### 4.5 Research advantages

First, the present study was conducted under controlled laboratory conditions with a rigorous experimental design and reliable data collection methods. This adds credibility to the study. Second, we examined the effects of vibration training from multiple perspectives by conducting a comprehensive analysis of the differences in energy expenditure between the REV and RE groups for different weight-bearing deep squat situations. In addition, the intervening factors in this study included both mechanical and extrinsic resistance load changes. This added to the complexity and scientific validity of the study. Finally, this study provides an efficient and easy-to-use exercise modality for the population wanting to lose fat. This provides practicality and guidance for real-world applications.

## 5 Conclusion

Whole-body vibration training may increase energy expenditure during low-intensity deep squat exercise and excess oxygen consumption within 30 min of exercise. However, when performing weighted squat training under vibration or non-vibration conditions within 40% of body mass, the energy consumption effect per unit of body weight is consistent with that caused by additional weight per kilogram carried. In addition, vibration training can be implemented through simple operations that improve our exercise efficiency through ease of use. The vibration stimulation of the wobbly platform also creates an adaptive stimulus that is psychologically conducive to withstanding greater training loads. However, increasing intensity requires caution and gradual progress. Therefore, in daily exercise activities, for people trying to lose fat through mass sports, if they are tired of regular running and weight training, they can try vibration training, through which the lean body mass and fat loss compared to the traditional means of exercise will be better.

## Data Availability

The original contributions presented in the study are included in the article/Supplementary Material; further inquiries can be directed to the corresponding author.
